# A novel method for the determination of organophosphorus pesticides in urine samples using a combined gas diffusion microextraction (GDME) and gas chromatography-mass spectrometry (GC–MS) technique

**DOI:** 10.1016/j.mex.2025.103212

**Published:** 2025-02-12

**Authors:** Mohammadreza Jafari, Ali Gholami, Maryam Akhgari

**Affiliations:** a Department of Analytical Chemistry, Faculty of Chemistry, University of Kashan, Kashan, Iran; b Legal Medicine Research Center, Legal Medicine Organization, Tehran, Iran

**Keywords:** Gas Diffusion Microextraction (GDME), Organophosphorus pesticides, Diazinon, Chlorpyrifos, Gas Chromatography-Mass Spectrometry (GC–MS), Forensic toxicology, Gas Diffusion Microextraction (GDME) combined with Gas Chromatography-Mass Spectrometry (GC–MS)

## Abstract

This study introduces a novel and sensitive method for determining organophosphorus pesticides in urine using Gas Diffusion Microextraction (GDME) combined with Gas Chromatography-Mass Spectrometry (GC–MS). The goal is to offer an efficient, cost-effective method for extracting and analyzing these toxic compounds, which are widely used and harmful to human health and the environment. Organophosphorus pesticides, such as diazinon and chlorpyrifos, are among the most toxic and prevalent. The study aims to validate a specific, sensitive sample preparation and detection method for diazinon in urine.

Urine samples from individuals not exposed to these pesticides were extracted with GDME, under optimal conditions of 60°C, 34 minutes, and 300 µL of receptor phase. Samples were analyzed using GC–MS. The method showed good linearity (0.01 to 100 µg/L) and excellent sensitivity with detection limits of 0.0058 µg/L for diazinon and 0.016 µg/L for chlorpyrifos.

• Results indicate the higher sensitivity and selectivity of GDME compared to traditional methods like solid-phase microextraction.
• GDME method for pesticide extraction demonstrated superior performance, with a much lower limit of detection for diazinon (0.0058 µg/L) than conventional methods (0.02 µg/L).
• This study highlights GDME's potential for accurate and reliable pesticide detection.

## Graphical abstract

**Figure:**
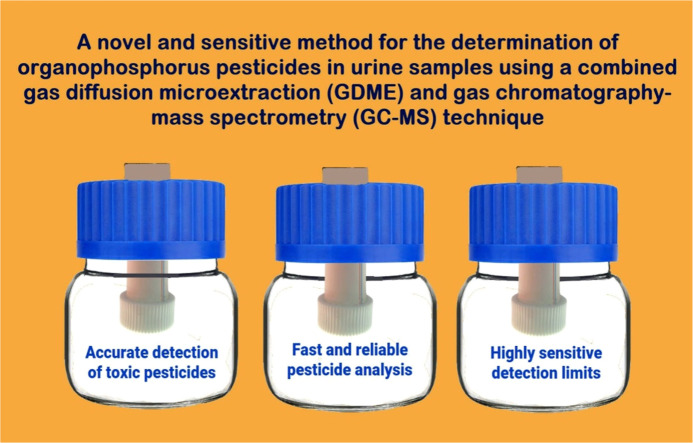

Specifications table

**Table:** 

Subject area:	Chemistry
More specific subject area:	• Determination of organophosphorus pesticide residues (such as diazinon and chlorpyrifos) in biological samples (urine) using advanced extraction methods • Gas diffusion microextraction (GDME) and gas chromatography-mass spectrometry (GC–MS) techniques
Name of your method:	Gas Diffusion Microextraction (GDME) combined with Gas Chromatography-Mass Spectrometry (GC–MS)
Name and reference of original method:	This study is the first to utilize the Gas Diffusion Microextraction (GDME) method for the extraction of organophosphorus pesticides. Key references related to the GDME and GC–MS extraction methods can be found in articles on gas-phase extraction techniques and GC–MS analysis.
Resource availability:	1) Equipment and Hardware: • Gas Chromatography and Mass Spectrometry (GC–MS): ○ Perkin Elmer Clarus 680 gas chromatograph ○ Perkin Elmer Clarus SQ 8 quadrupole mass spectrometer ○ Auto-sampler device • Chromatographic Column: 30 m × 0.25 mm I.D. with a 0.25 µm film thickness (HP-5 MS), from J & W Scientific • Carrier Gas: Helium at a flow rate of 1.0 mL/min • Ionization Conditions: Electron Impact (EI) mode with an energy of 70 eV and filament current of 35 µA 2) Software: Design and Data Analysis Software: Design-Expert 13 for data analysis and experimental design Link to Design-Expert software: Design-Expert 13 Data Acquisition Software: Perkin Elmer TotalChrom for GC–MS data 3) Chemicals: Pesticides for the study: Diazinon (DZN) and Chlorpyrifos (CPF) from Sigma-Aldrich, St. Louis, MO, USAMalathion (Internal Standard) from Sigma-Aldrich, St. Louis, MO, USAMalathion was selected as the internal standard for its chemical similarity to diazinon and chlorpyrifos, sharing functional groups and similar behavior during GDME extraction and analysis. Its well-separated chromatographic peak avoids interference with analytes, ensuring precise quantification. Malathion's high stability under analytical conditions prevents degradation, maintaining result accuracy. It compensates for matrix effects by minimizing signal variations due to sample complexity, enhancing reliability. Malathion demonstrates consistent recovery across biological matrices, aligning closely with the behavior of target analytes. Its compatibility with vapor-phase extraction ensures effective transfer during GDME, and its lower toxicity reduces safety concerns in experimental. Solvents: Methanol and Acetonitrile from Merck, Darmstadt, Germany

## Background

The motivation for developing and optimizing the method presented in this study is based on the need for a sensitive, reliable, and efficient approach to detecting organophosphorus pesticides such as diazinon and chlorpyrifos in biological samples, particularly urine. Organophosphorus pesticides are commonly used in agriculture and are associated with significant health risks due to their neurotoxic effects [[1], [2], [3]]. Detecting trace amounts of these pesticides in biological matrices is essential for monitoring exposure, especially in forensic toxicology, where accurate analysis is critical for determining cause of poisoning and exposure levels [[4], [5], [6], [7]].

Traditional methods for pesticide detection, such as solid-phase microextraction (SPME) or liquid-liquid extraction (LLE), often face challenges due to sample complexity and the need for high sensitivity at low concentrations. These methods can also be time-consuming, involve hazardous solvents, or suffer from issues such as matrix interferences and poor reproducibility [[1], [5], [8], [9], [10], [11], [12], [13], [14], [15]]. This study introduces a novel gas diffusion microextraction (GDME) technique combined with gas chromatography-mass spectrometry (GC–MS) to address these challenges.

One of the primary advantages of the GDME method is its ability to efficiently extract organophosphorus compounds from complex biological matrices, such as urine, with minimal sample preparation. The method is designed to reduce interferences from matrix components such as proteins, salts, and urea, which commonly complicate pesticide extraction from biological samples. The use of dilution and an internal standard, like malathion, effectively mitigates these interferences, ensuring accurate pesticide quantification even at low concentrations [[4], [9], [10]]. By improving the selectivity and sensitivity of the extraction process, GDME enhances the reliability of the measurements and increases the method's applicability in forensic toxicology.

The Box-Behnken Design (BBD), a Response Surface Methodology, was chosen for its ability to optimize extraction conditions with minimal experiments and high accuracy [16]. It requires fewer experiments than Central Composite Designs (CCD), focusing on optimal variable ranges without extreme values, reducing costs and time. BBD efficiently analyzes nonlinear interactions and complex relationships between key variables like extraction time, temperature, and acceptor solvent volume, enabling comprehensive modeling. Its simplicity, user-friendly implementation, and compatibility with statistical software make it ideal for method optimization. Compared to other techniques, BBD outperforms One-Variable-at-a-Time designs by evaluating variable interactions, avoids the impractical exponential experiment growth of Full Factorial Designs, and is more efficient than CCD, which demands more experiments due to corner points [17].

Furthermore, the optimization of key extraction parameters—such as time, temperature, and solvent volume—using the Box-Behnken Design (BBD) has resulted in the determination of optimal conditions for maximizing extraction efficiency. These conditions, including a 34-minute extraction time at 60°C using a solvent volume of 300 µL, significantly enhance the recovery and concentration of pesticides, which is crucial for detecting low levels of organophosphorus compounds in biological samples [[1], [9], [16]].

The GDME method, with its rapid solvent evaporation and high solubility of methanol and acetonitrile, also improves separation efficiency by reducing interferences with analyte peaks. This allows for the precise and reproducible quantification of pesticides, even in challenging matrices like urine. The combination of optimization strategies and advanced analytical techniques, like GC–MS, positions this method as a powerful tool for pesticide detection, offering several advantages over traditional methods, including higher sensitivity, reduced sample preparation time, and enhanced reliability [[11], [12], [13], [18]].

In conclusion, the methodology presented here offers a promising solution for the accurate and efficient determination of organophosphorus pesticides in forensic and clinical settings, where rapid, reliable, and cost-effective detection is paramount. The method's ability to handle complex biological matrices while minimizing interference makes it a valuable addition to the current toolbox for pesticide monitoring and exposure assessment.

## Method details

### Technical Steps for the GDME Extraction Method

For the GDME extraction method, optimization of extraction conditions including temperature, time, and solvent volume was carried out. Special attention was given to various parameters, such as temperature and the contact time between the sample and the extraction solvent, to achieve the best extraction efficiency.

**Fig. 1:**
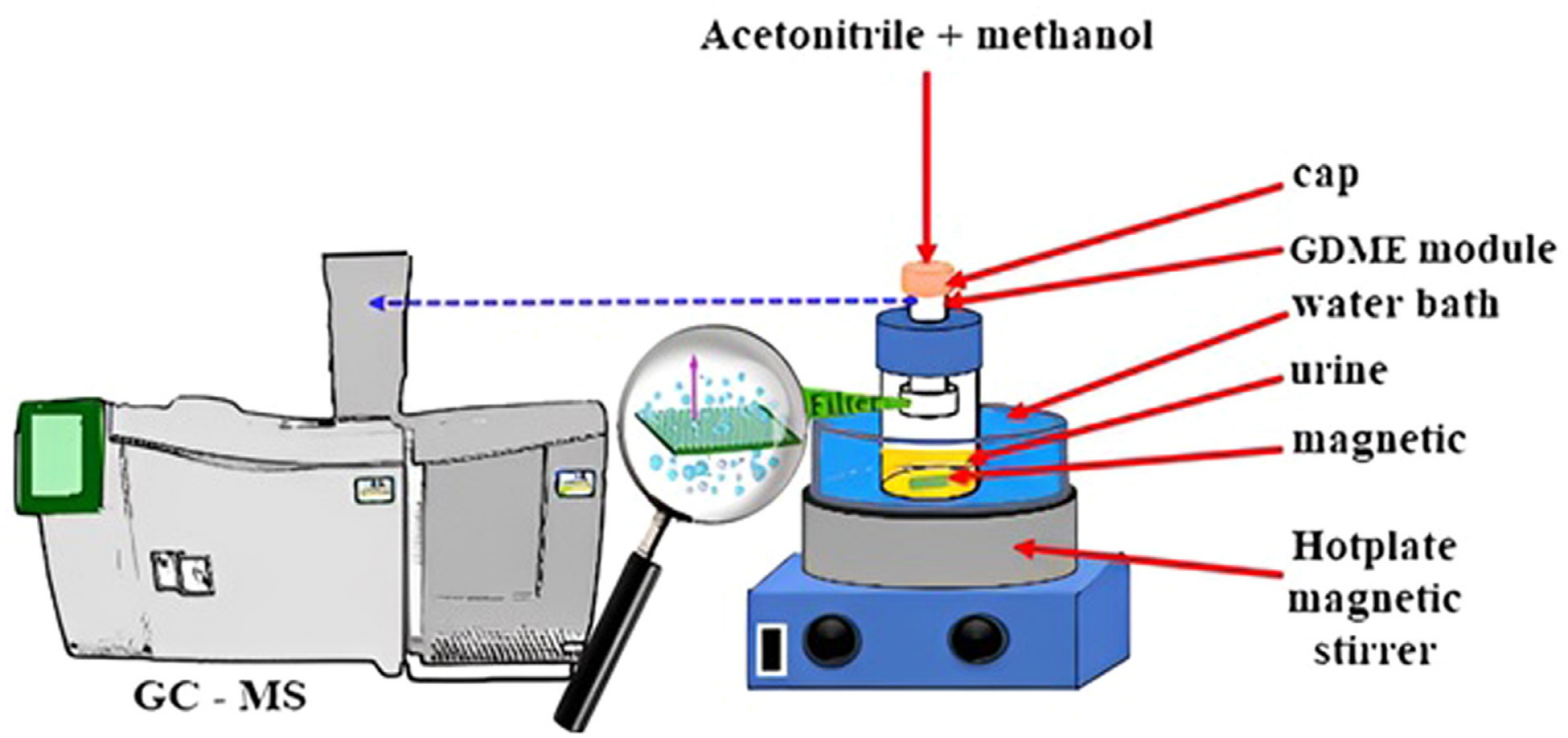
Experimental method design for the detection of organophosphorus compounds in urine specimen.

Procedure Guidance (Fig. 1):

1. First, a specified volume of urine sample is transferred to the GDME device.
2. After setting the optimized conditions for temperature and time (previously determined through experiments), the extraction solvent is introduced.
3. At this stage, it is critical to control the volume of the solvent and the contact time to ensure efficient analyte transfer to the vapor phase.
4. After the extraction process is completed, the samples are prepared for analysis using Gas Chromatography-Mass Spectrometry (GC–MS).

Additional Observations and Tips:

• Choosing the right solvent and precisely controlling its volume significantly impacts the accuracy of the results.
• Lower temperatures in some cases may enhance extraction efficiency.
• To minimize matrix interferences, using smaller volumes of solvent is recommended.

The methanol-acetonitrile mixture was selected as the receiving phase in the Gas Diffusion Microextraction (GDME) method for its superior performance in capturing diazinon and chlorpyrifos. The mixture enhances analyte solubility, with methanol offering high polarity and acetonitrile providing stable, semi-polar compatibility. It optimizes absorption efficiency by enabling rapid analyte stabilization while minimizing re-evaporation due to its low viscosity and volatility. The mixture also reduces matrix interferences from biological components, improving sensitivity for complex samples like urine. Its compatibility with GC–MS ensures residue-free evaporation, sharp chromatographic peaks, and accurate analysis. By combining the strengths of methanol and acetonitrile, their limitations are mitigated, resulting in high analyte recovery, reduced interference, and enhanced method reliability.

This met demonstrates superior performance in comparison to other methods, such as Solid-Phase Extraction (SPE) and Liquid-Liquid Microextraction (LLME), in terms of higher extraction efficiency and precision. The experimental results show that the GDME method offers better recovery rates and greater accuracy than other extraction techniques.

### Method validation

For method validation, the accuracy and precision of the extraction were assessed using different biological samples (urine). The data provided below substantiate the value of the GDME/GC–MS method for the extraction and quantification of diazinon and chlorpyrifos in biological samples.

1. **Calibration Curves and Linearity**: After optimizing extraction conditions, calibration curves for diazinon and chlorpyrifos were constructed over a concentration range of 0.01 to 100 µg/L. The curves exhibited excellent linearity, with coefficients of determination (R²) greater than 0.995, indicating a strong and direct correlation between analyte concentration and signal intensity.
2. **Limit of Detection (LOD) and Limit of Quantification (LOQ)**: The LOD and LOQ values, calculated using standard formulas and the signal-to-noise ratio method, demonstrate remarkable sensitivity. For diazinon and chlorpyrifos, the LODs were determined as 0.0058 µg/L and 0.019 µg/L, respectively, while the LOQs were 0.019 µg/L and 0.063 µg/L. These values are significantly lower than legal thresholds and reported poisoning levels. The LOQ for diazinon is well below the acute poisoning threshold (10 µg/L) and regulatory limits (0.01–0.1 mg/kg), while that for chlorpyrifos is far beneath clinical poisoning concentrations (20–100 µg/L) and acceptable limits (<10 µg/L). This exceptional sensitivity allows the method to detect both acute poisoning (concentrations >100 µg/L) and chronic exposure (below 10 µg/L). The proposed GDME-GC–MS method reliably detects and quantifies low pesticide levels associated with chronic exposure, with its sensitivity surpassing conventional techniques like SPME or SBSE. Additionally, its accuracy and precision at low concentrations make it particularly effective for chronic poisoning cases. This method enables routine monitoring in complex biological samples, such as urine, providing reproducible results with high efficiency and speed. As a result, the GDME-GC–MS technique emerges as a highly sensitive and dependable tool for biomonitoring and detecting diazinon and chlorpyrifos at levels well below legal and toxic thresholds, making it ideal for both routine and forensic applications.
3. **Repeatability**: Intra-day and inter-day relative standard deviations (RSDs) were calculated for diazinon and chlorpyrifos at various concentrations (0.05, 5, and 50 µg/L). The intra-day and inter-day RSDs were less than 1.31% and 1.83%, respectively, demonstrating excellent reproducibility of the method.
4. **Extraction Recovery**: Extraction recoveries were calculated to assess the efficiency of the method. For both analytes, high extraction recoveries were observed—99.02% for diazinon and 98.86% for chlorpyrifos—demonstrating the high precision of the GDME method.
5. **Comparison with Other Methods**: The GDME/GC–MS method was compared with other commonly used extraction methods such as solid-phase extraction (SPE), liquid-liquid microextraction (LLME), and disposable pipette extraction (DPX). The GDME/GC–MS method showed superior performance in terms of LOD, LOQ, precision, and extraction efficiency. For example, the GDME method demonstrated a lower LOD and higher recovery rates compared to the SPE and LLME methods.
6. **Extraction Efficiency**: The extraction efficiency achieved with the GDME/GC–MS method was 99.0% for diazinon and 98.9% for chlorpyrifos. This is significantly higher than extraction efficiencies reported for other methods, such as SPE (96%) and LLME (69%).
7. **Statistical Analysis**: ANOVA analysis confirmed that the optimized parameters (temperature, time, and solvent volume) significantly impacted the extraction efficiency, further supporting the validity of the method. Analysis of Variance (ANOVA) evaluated the significance of extraction time, temperature, and acceptor solvent volume on extraction efficiency, revealing that each parameter significantly influenced analyte transfer to the acceptor phase. Longer extraction times improved transfer but risked saturation or degradation, while higher temperatures enhanced transfer rates, and optimal solvent volumes balanced retention and dilution. Interaction analysis showed that temperature and time had the most significant combined effect, optimizing membrane permeability, while large solvent volumes at high temperatures reduced efficiency due to dilution. Statistical metrics like high F-values, significant p-values, and R² = 0.98 confirmed the model's predictive accuracy. ANOVA identified key factors for optimization, reduced experimental costs, and improved accuracy. Manuscript revisions include expanded explanations, enhanced tables and graphs, and detailed discussion of parameter effects and ANOVA outcomes.
8. **Environmental Impact**: The GDME method is recognized as more environmentally friendly compared to solvent-based extraction methods like LLME, which face issues related to contamination and waste disposal. The reduced use of organic solvents and smaller sample volumes makes GDME a greener alternative.

These findings validate the GDME/GC–MS method as a reliable, sensitive, and efficient technique for the extraction and analysis of organophosphorus pesticides, particularly in biological matrices. The method offers significant advantages over conventional techniques in terms of sensitivity, repeatability, and environmental sustainability, making it a highly suitable choice for researchers and practitioners.

## Results and discussion

### Optimization methods for extracting and analyzing organophosphorus compounds from biological samples

**Reducing Interferences:**

Choosing optimal extraction conditions for diazinon and chlorpyrifos from urine using the GDME method is essential for ensuring accurate and reliable results. The presence of interfering compounds such as proteins, urea, and salts in urine samples can present challenges in achieving precise extraction of organophosphorus compounds [9]. These compounds may interact with the analytes, leading to reduced accuracy and precision in measurements [[11], [17]]. To mitigate these interferences, diluting the urine sample is an effective approach. Dilution decreases the concentration of interfering compounds, making their separation easier. As a result, the complexity of the sample matrix is reduced, enabling more accurate measurement of pesticides at low levels in urine. Studies have shown that diluting samples can improve the accuracy of pesticide measurements at low concentrations [19]. Another effective strategy for reducing interferences in complex samples like urine is the use of an internal standard. By employing an internal standard, variations caused by the complex urine matrix affect both the internal standard and the target compound equally, maintaining a relatively constant ratio between their signals. The internal standard should be chemically similar to the target compound so that it is affected similarly by the sample matrix. In this study, malathion was chosen as the internal standard. Malathion is an organophosphorus compound that is structurally similar to diazinon and chlorpyrifos.

**Selection of Appropriate Solvent:**

The selection of a suitable solvent is another critical factor in the extraction of organophosphorus compounds from urine. The combination of methanol and acetonitrile is considered ideal for this purpose due to its high solubility and appropriate volatility [20]. The rapid evaporation of these solvents after extraction results in the concentration of pesticides in the extract and reduces interferences with analyte peaks. Therefore, the use of this solvent mixture as the acceptor phase can significantly enhance separation efficiency and improve measurement accuracy.

**Two-Stage Extraction:**

Two-stage extraction is an effective strategy for improving extraction efficiency. In the first stage, using the module cap helps maintain pressure and temperature within the system, which aids in accelerating mass transfer. This process is similar to Total Vaporization Solid-Phase Microextraction (TV-SPME) and fully transfers the target compounds from the sample matrix into the gas phase [21]. Additionally, the cap in the first stage prevents the unwanted evaporation of solvents from the module. In the second stage, by removing the cap, the system allows for equilibrium between the solvent and sample phases, facilitating the partial evaporation of the solvent and concentrating the pesticide-containing extract. This concentration in the second stage (without the cap) increases the sensitivity of the GC–MS analysis, enabling the detection of lower pesticide concentrations in urine.

**Optimization of Extraction Parameters Using the Box-Behnken Design:**

Recent research has highlighted the crucial role of parameters such as time, temperature, and solvent volume in the efficiency of GDME [22]. The Box-Behnken Design (BBD) is an effective tool for optimizing these factors in experiments, allowing for a detailed examination of both their individual and interactive effects. In this study, BBD with 15 experiments was employed to optimize the extraction of diazinon at a concentration of 1 µg/L. The values of each factor and the corresponding responses are presented in Table 1, with each response being the average of three replicates under the same conditions. Diazinon and chlorpyrifos share many chemical similarities, so the optimized conditions for diazinon may also be applicable for chlorpyrifos.

**Table 1:** Box-Behnken Design for Optimizing Factors Affecting Diazinon Extraction.

Standard Run	Run	Time (minutes)	Temperature (**°C**)	Volume (µL)	Surface area under the peak
2	1	40	40	500	469,863
13	2	30	50	500	680,110
8	3	40	50	700	431,379
15	4	30	50	500	700,312
12	5	30	60	700	658,304
5	6	20	50	300	769,845
6	7	40	50	300	887,214
11	8	30	40	700	386,273
7	9	20	50	700	421,123
4	10	40	60	500	737,321
10	11	30	60	300	946,396
14	12	30	50	500	690,211
3	13	20	60	500	609,875
9	14	30	40	300	823,492
1	15	20	40	500	469,863

To evaluate the suitability of the Box-Behnken design model and analyze the data, we used criteria and results from analysis of variance (ANOVA), which helped us understanding how well the model fits the data and meets the analytical needs. Table 2 presents the ANOVA results for the BBD model and the significance of the effects of factors A (time), B (temperature), and C (volume), as well as their interactions on the peak area of diazinon. The overall model significantly explains the experimental data, with an F value of 240.27 and a p-value less than 0.0001. Among the main factors, temperature (B) and volume (C) had the most significant effects on the response, with F values of 398.94 and 1450.20 and p-values less than 0.0001, respectively. Additionally, the interaction effects AB, AC, and BC were significantly influential, indicating the importance of interactions between these factors. The quadratic effects (A², B², and C²) were also significant, emphasizing the need to consider nonlinear effects in optimizing extraction conditions. The lack of fit test was not significant (p-value = 0.2875), indicating a good fit of the model to the experimental data.

**Table 2:** Analysis of Variance for Box-Behnken Design.

Model	4.363E+11	9	4.847E+10	240.27	< 0.0001	significant
A-time	8.133E+09	1	8.133E+09	40.31	0.0014	
B-temperature	8.048E+10	1	8.048E+10	398.94	< 0.0001	
C-volume	2.926E+11	1	2.926E+11	1450.20	< 0.0001	
AB	4.061E+09	1	4.061E+09	20.13	0.0065	
AC	2.868E+09	1	2.868E+09	14.22	0.0130	
BC	5.560E+09	1	5.560E+09	27.56	0.0033	
A²	3.499E+10	1	3.499E+10	173.46	< 0.0001	
B²	1.648E+09	1	1.648E+09	8.17	0.0355	
C²	4.403E+09	1	4.403E+09	21.83	0.0055	
Residual	1.009E+09	5	2.017E+08			
Lack of Fit	8.046E+08	3	2.682E+08	2.63	0.2875	not significant
Pure Error	2.041E+08	2	1.020E+08			
Cor Total	4.373E+11	14				

These results indicate that the optimal conditions for diazinon extraction using the Box-Behnken design have been effectively determined and may also be suitable for the separation of chlorpyrifos due to the chemical structural similarity between the two compounds.

The optimal values for time, temperature, and volume were determined to be 34 minutes, 60°C, and 300 µL, respectively. These values appear to produce the maximum area under the curve. The predicted area under the curve using the optimal values is 976,560, which is significantly higher than the range of observed values (minimum of 386,273 and maximum of 946,396). This indicates the success of the optimization in achieving the goal of maximizing the area under the curve. The validity of the predictions is assessed using confidence and prediction intervals. The small standard error of prediction and the reasonably narrow confidence and prediction intervals indicate the method's capability to accurately explain and predict the response (diazinon extraction yield).

**Fig. 2:**
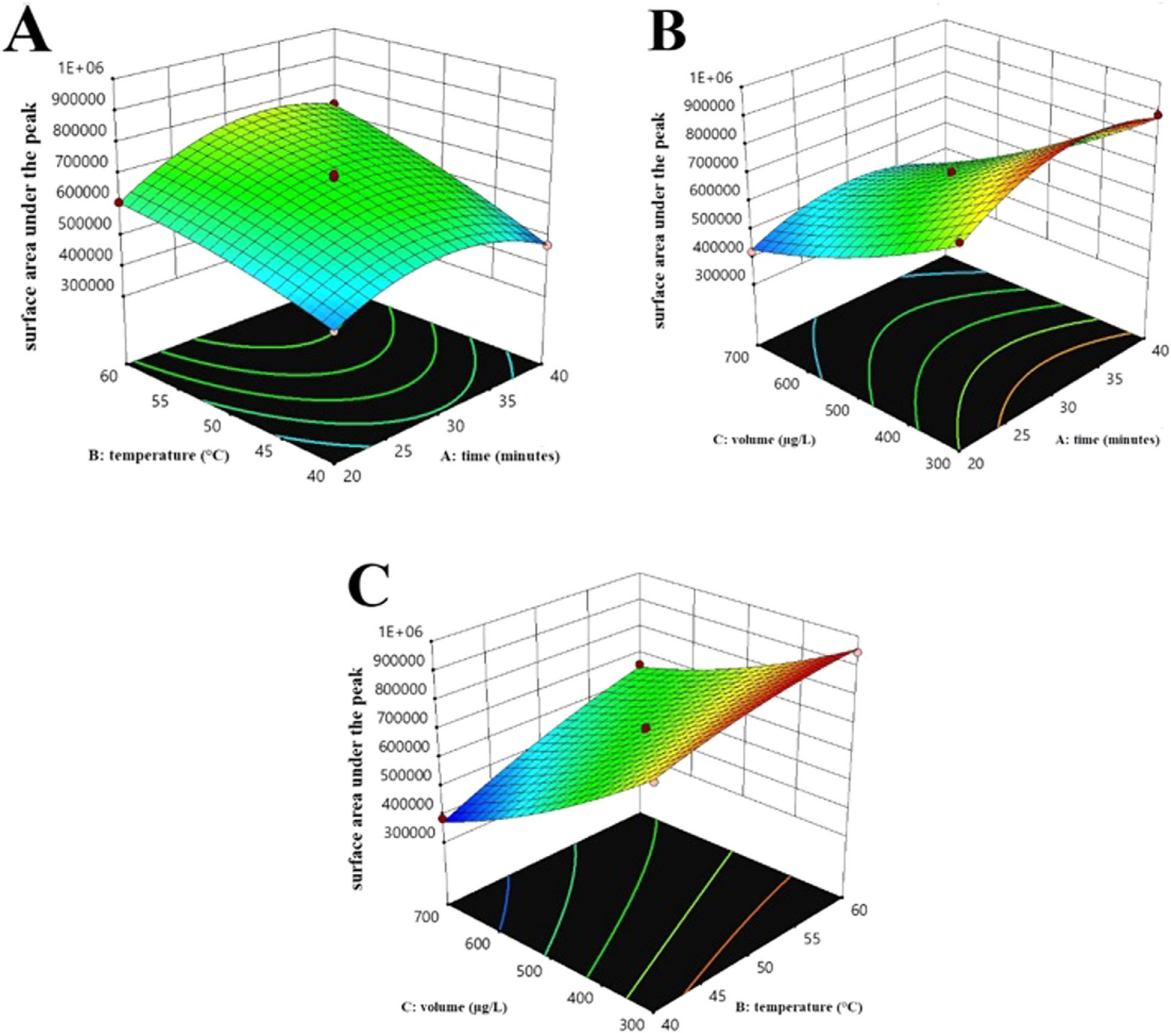
Three-dimensional response surface plots of the effects of two factors on diazinon extraction efficiency. A: Interaction effect of time and temperature on diazinon extraction efficiency. B: Interaction effect of time and volume on diazinon extraction efficiency. C: Interaction effect of temperature and volume on diazinon extraction yield.

The three-dimensional response surface plots presented in Fig. 2 illustrate the results of the optimization experiments using the Box-Behnken design. These plots visually represent the relationships between the independent variables (time, temperature, and volume) and the response variable (peak area). Fig. 2A shows the effect of time and temperature on the peak area. As observed, simultaneous increases in time and temperature lead to a significant increase in the peak area. This indicates a positive impact of both variables on the extraction efficiency of diazinon. The optimal point in this plot is near 30 minutes and 60°C, suggesting a favorable balance between the rate of mass transfer reactions and the prevention of target material degradation. The positive interaction between time and temperature also implies that the effect of increasing temperature is more pronounced at longer times. Fig. 2B depicts the effect of time and volume on the peak area. Increasing the solvent volume slightly increases the peak area, but this increase is not as significant as that observed with time and temperature. The optimal point in this plot is near 300 microliters, indicating that excessive solvent volume does not significantly impact extraction efficiency. A weak negative interaction between time and volume is also observed. Fig. 2C illustrates the effect of temperature and volume on the peak area. Simultaneous increases in temperature and volume result in an increase in the peak area, but this increase is not as substantial as that observed in Fig. 2A. The optimal point in this plot is near 60°C and 300 microliters, indicating a suitable balance between temperature, volume, and extraction efficiency. A positive interaction between temperature and volume is also observed.

High temperature (60°C) significantly positively impacts the mean response (diazinon extraction rate). As temperature increases, the kinetic energy of solvent molecules rises, causing them to move more rapidly and thereby reducing the intermolecular forces. Consequently, the viscosity of the extracting solvent decreases, which facilitates diazinon diffusion and increases collisions between diazinon and solvent molecules. Therefore, with higher temperature, diazinon solubility in the extracting solvent increases, accelerating the extraction process and enhancing extraction efficiency. However, further temperature increases also raise the risk of thermal degradation of diazinon, potentially reducing the concentration of extractable diazinon in the sample. A time of 34 minutes is identified as optimal for achieving high yield. Adequate time is crucial for completing the extraction process. In the initial stages of extraction, longer times can improve extraction efficiency as the solvent has sufficient opportunity to contact the sample and transfer diazinon to the solvent phase. However, once equilibrium between the solvent and sample phases is reached, extending the extraction process does not enhance yield and may even cause the extracted materials to revert to the original phase or result in diazinon degradation, especially under high thermal conditions. This degradation can lead to a decrease in the extractable diazinon concentration. Solvent volume plays a critical role in the diazinon extraction process. A volume of 300 microliters demonstrated the best yield compared to other volumes. Reducing the volume increases the diazinon concentration in the solution due to the decreased space for diazinon molecules, which raises their concentration in the solution. Additionally, reducing solvent volume can improve the contact surface between the solvent and sample, providing more opportunities for diazinon molecules to transfer from the sample phase to the solvent phase, thus increasing extraction yield. In contrast, increasing solvent volume dilutes the solution, reducing diazinon concentration and decreasing the balance between diazinon in the sample and solvent phases, leading to reduced extraction yield. Furthermore, excessively high solvent volumes significantly decrease the diazinon-to-solvent ratio, dramatically lowering extraction yield. The choice of an appropriate solvent is also a key factor in the extraction of phosphorus-containing organic compounds from biological samples. A combination of methanol and acetonitrile is considered ideal due to its high solubility and appropriate volatility. The rapid evaporation of these solvents after extraction increases the concentration of pesticides in the extract and reduces interference with analyte peaks. Therefore, using this solvent mixture as the receiving solution can significantly improve separation and increase measurement accuracy.

**Comparison of Optimization with Other Extraction Methods:**

Extraction temperature is a key parameter in vapor-phase extraction methods such as GDME, SPME, SBSE, and SDME. Increasing the temperature generally leads to an increase in the vapor pressure of the analytes, which facilitates their transfer to the extraction phase. In the proposed GDME method, the optimal extraction temperature was determined to be 60°C, providing a balance between the increased vapor pressure of the analytes and the stability of the membrane. This temperature effectively enhances the diffusion rate of diazinon and chlorpyrifos from the urine sample to the receiving phase.

In comparison with other methods, solid-phase microextraction (SPME), stir bar sorptive extraction (SBSE), and single-drop microextraction (SDME) have also experienced similar temperature optimizations. For example, in SPME, temperatures between 40 and 70°C have been reported as optimal for the extraction of semi-volatile pesticides [14]. In SBSE, higher temperatures up to 80°C have been explored, although such increases may lead to the degradation or loss of unstable compounds [23]. In SDME, an optimal temperature of 60°C was identified during optimization [24]. Generally, the results indicate that exceeding a certain temperature threshold may result in a loss of extraction selectivity, and temperature optimization is critical depending on the analytes and extraction configuration used.

In the GDME method, neutral pH was selected as the optimal condition, as this method exhibits low sensitivity to pH variations. This means that diazinon and chlorpyrifos, the compounds studied in this research, dissolve well under neutral pH conditions, leading to enhanced extraction efficiency. Studies have shown that neutral pH preserves the non-ionic form of the compounds, thereby increasing their solubility and extractability [25].

### Chromatographic analysis

**Fig. 3:**
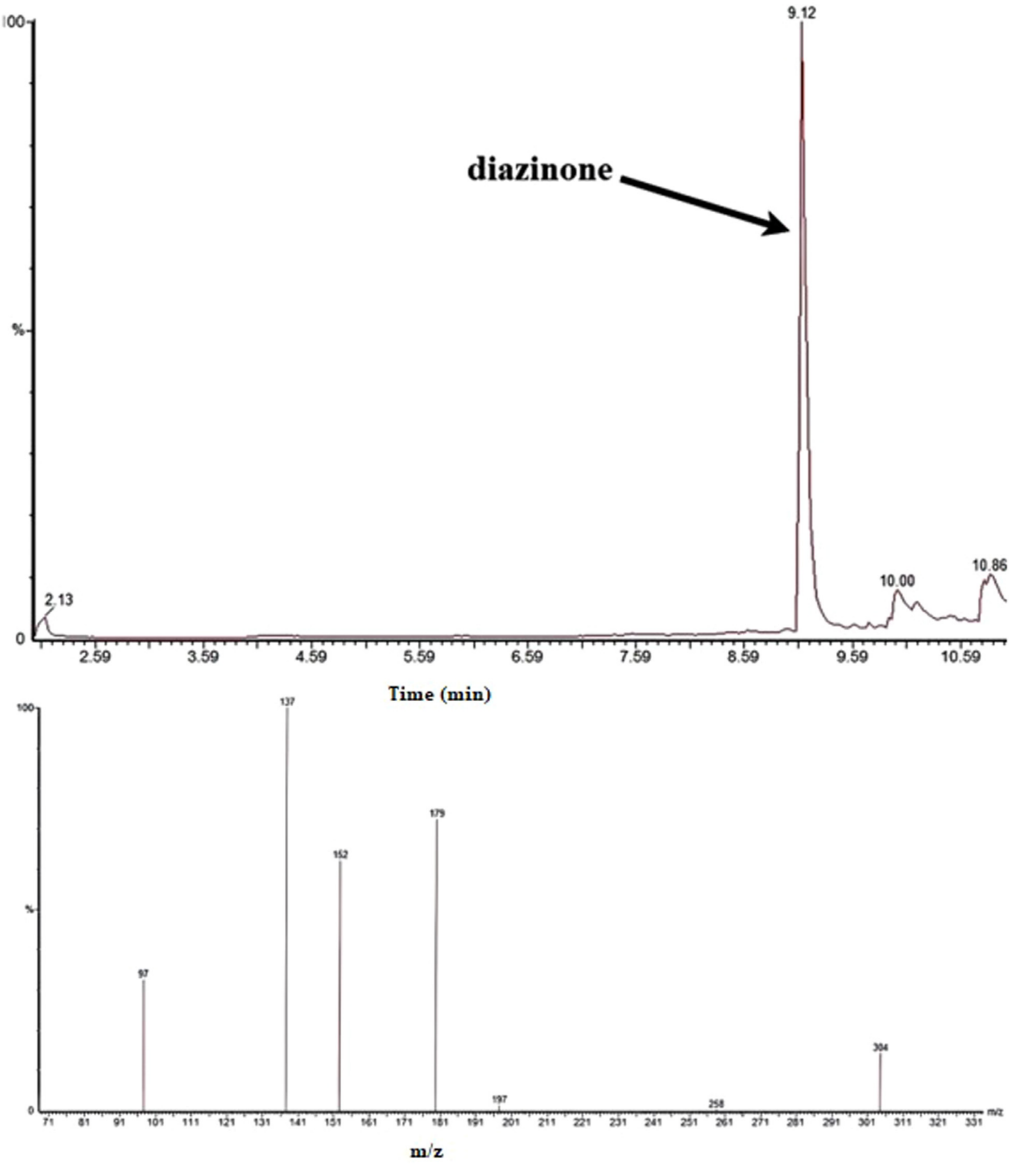
Chromatogram and spectrum obtained from HS-GDME/GC–MS analysis of urine sample for diazinon (1 µg/L) in SIR mode.

**Fig. 4:**
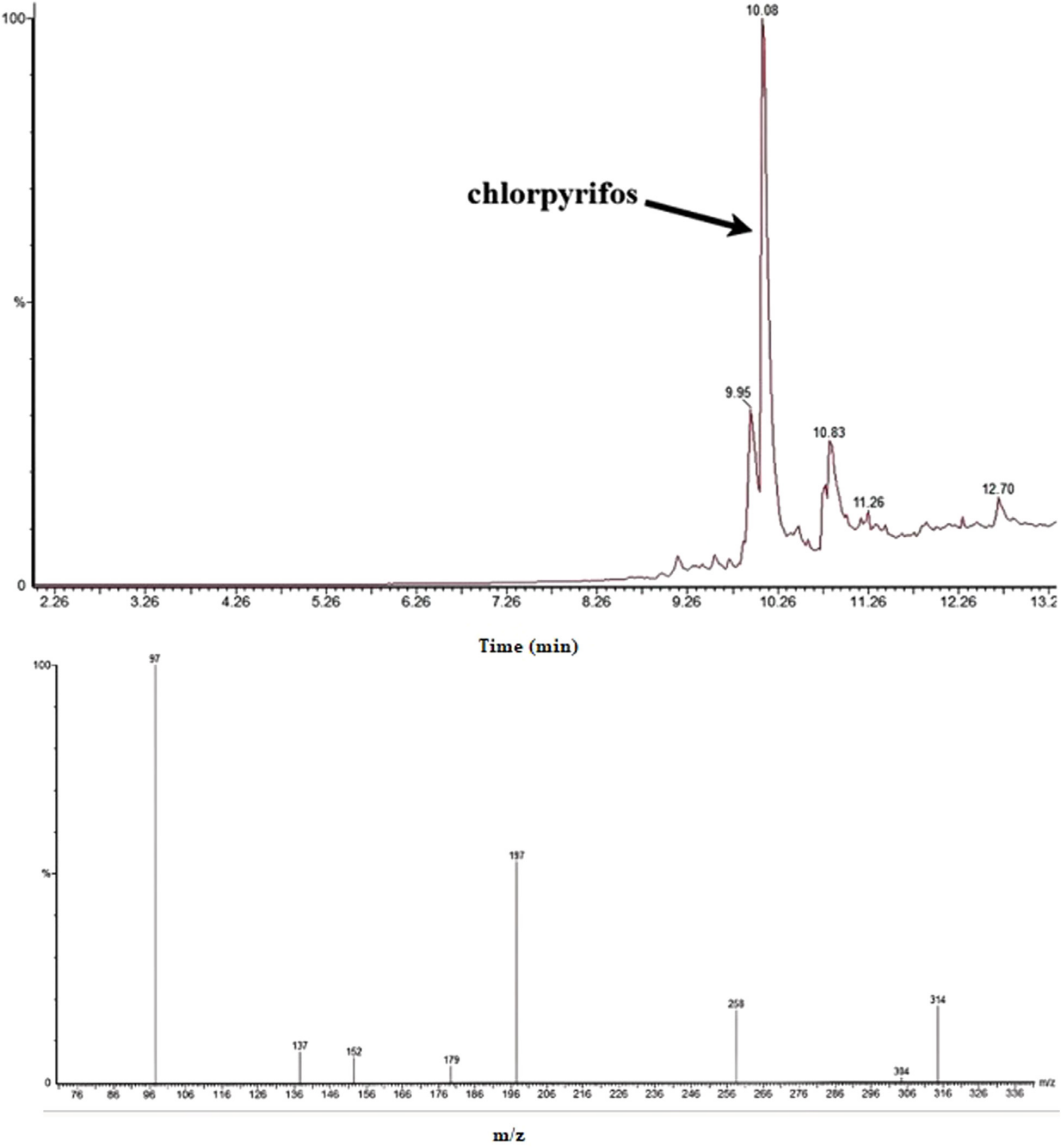
Chromatogram and spectrum obtained from HS-GDME/GC–MS analysis of urine sample for chlorpyrifos (1 µg/L) in SIR mode.

**Fig. 5:**
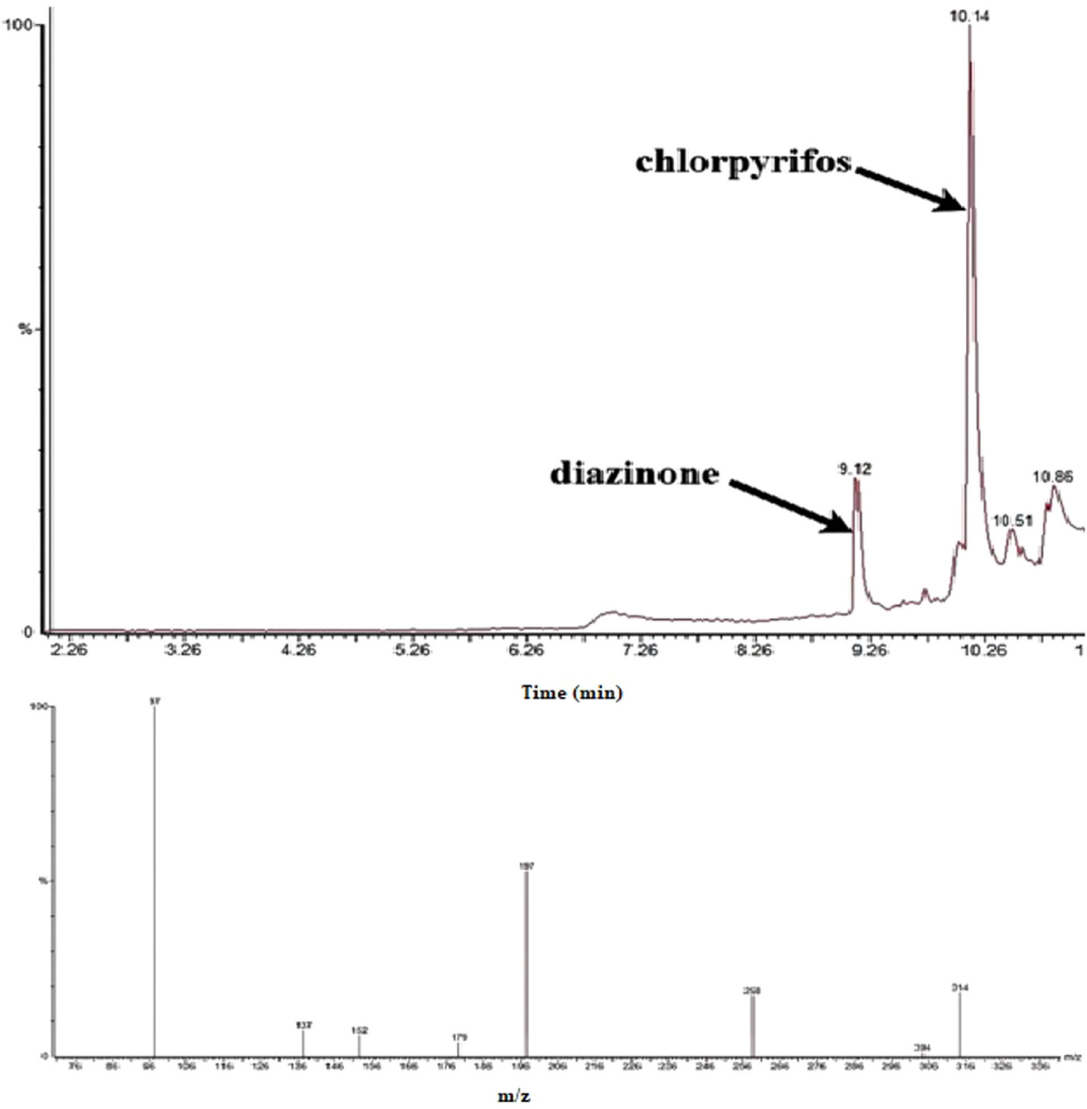
Chromatogram and spectrum obtained from HS-GDME/GC–MS analysis of urine sample for diazinon and chlorpyrifos (1 µg/L) in SIR mode.

The chromatographic results obtained using the GC–MS system (Fig. 3, Fig. 4, Fig. 5) demonstrate complete and accurate separation of diazinon and chlorpyrifos in urine samples. Sharp and symmetrical peaks for these two compounds were observed at retention times of 9.12 and 10.14 minutes, respectively. The identity of these compounds was confirmed by comparing the mass spectra obtained with standard NIST libraries, ensuring full matching of molecular ions and characteristic fragment ions. Furthermore, the optimized extraction conditions using GDME have enhanced the sensitivity and selectivity of the chromatographic method, enabling precise detection of these compounds at low levels. This has played a crucial role in improving the separation and accurate identification of diazinon and chlorpyrifos in the chromatograms. The mass spectra obtained for both compounds fully matched the reference standards, and the presence of characteristic molecular ions at [m/z values] and fragment ions at [m/z values] confirmed the presence of these compounds in the samples. These results indicate that the proposed method exhibits high sensitivity and selectivity for the quantitative and qualitative determination of diazinon and chlorpyrifos in biological samples. A comparison of the chromatograms for diazinon and chlorpyrifos shows that these two compounds were separated by different retention times and peak patterns. The difference in retention times is attributed to differences in molecular structure and varying interactions with the stationary phase of the column. Additionally, differences in the intensity of characteristic ion peaks in the mass spectra have aided in the accurate identification of each of these compounds.

### Analytical parameters and sample analysis

After optimizing the extraction conditions using the GDME method, calibration curves for diazinon and chlorpyrifos were constructed over a concentration range of 0.01 to 100 µg/L. These curves demonstrated excellent linearity with a coefficient of determination (R²) greater than 0.995, indicating a strong and direct relationship between the analyte signal and its concentration.

Limit of Detection (LOD) and Limit of Quantitation (LOQ) are defined as the lowest concentrations at which an analyte can be reliably detected and quantified, respectively. The LODs and LOQs for an analytical method were calculated using the following equations [22]:

$$\text{LOD}=3S_{b}/m$$

$$\text{LOQ}=10S_{b}/m$$

where S_b_ and m represent the standard deviation of the blank solution and the slope of the calibration curve, respectively. The LODs and LOQs were found to be less than 0.0058 and 0.019 µg/L for diazinon, and less than 0.016 and 0.054 µg/L for chlorpyrifos (Table 3). The mean intra-day and inter-day RSDs for both analytes were less than 1.31 % and 1.83 %, respectively. Extraction recoveries were also calculated using the ratio of the analyte concentration before and after extraction according to the following equation:

$$\%\text{ER}=\left(C_{a}/C_{b}\right)\times 100$$

where C_a_ and C_b_ represent the analyte concentration after extraction and in the sample solution before extraction, respectively. High extraction recoveries (99.02 % for diazinon and 98.86 % for chlorpyrifos) indicate the high precision of the method in extracting and quantifying these compounds. These results are compared with previous studies presented in Table 5, demonstrating the superiority of the proposed method.

**Table 3:** Quantitative Results of the GDME-GC–MS Method for Diazinon and Chlorpyrifos.

Analyte	Linear range (µg/L^-1^)	R^2^^a^	LOD^b^ (µg/L^-1^)	LOQ^c^ (µg/L^-1^)	%ER^d^±SD
Diazinon	0.01–100	0.9988	0.0058	0.019	99.024±5.75
Chlorpyrifos	0.01–100	0.9985	0.016	0.054	98.86±1.83

a Coefficient of determination

b LOD, limit of detection for 3S_b_/m

c LOQ, limit of quantitation for 10S_b_/m

d Extraction recovery

For method reproducibility, the relative standard deviation (RSD) was evaluated for the lowest, average, and highest concentrations measured, namely 0.05, 5, and 50 µg/L, respectively, over one day (intra-day RSD) or three consecutive days (inter-day RSD) (Table 4). The intra-day and inter-day RSD values were less than 1.31 % and 1.83 %, respectively, indicating excellent reproducibility of the method.

**Table 4:** Intra-Day (%) and Inter-Day (%) Precision at Three Concentration Levels for Diazinon and Chlorpyrifos.

Analyte	RSD% (n=5)					
	inter-day			intra-day		
	0.05 (µg/L^-1^)	5 (µg/L^-1^)	50 (µg/L^-1^)	0.05 (µg/L^-1^)	5 (µg/L^-1^)	50 (µg/L^-1^)
Diazinon	2.8	1.3	0.4	3.2	1.3	1.0
Chlorpyrifos	2.00	1.40	0.47	2.67	1.60	1.16

Analysis of variance (ANOVA) results showed that all optimized parameters (temperature, time, and solvent volume) had a significant impact on extraction efficiency. These results confirm that the selected optimal conditions for extraction enhance the sensitivity and accuracy of the method.

### Comparison with Other Extraction Methods

The results obtained from the proposed GDME/GC–MS method were compared with similar studies that employed various extraction techniques such as solid-phase extraction (SPE), liquid-liquid microextraction (LLME), and disposable pipette extraction (DPX) (Table 5). As shown in the table, the GDME/GC–MS method demonstrates a lower limit of detection (LOD) and limit of quantification (LOQ), a wider linear range, a higher coefficient of determination (R²), and greater precision and repeatability compared to other methods. For instance, the SPE method generally exhibits lower sensitivity and a narrower linear range compared to GDME. Additionally, solvent-based methods like LLME may face challenges such as contamination and wastewater disposal. In contrast, GDME is a greener, more environmentally friendly method due to the reduced use of organic solvents and smaller sample volumes.

**Table 5:** Comparison of the Proposed Method with Other Extraction Techniques for Analyzing Target Analytes in Urine Samples.

Row	Sample preparation	Pesticides	DLR^a^	LOD	LOQ	R2	RSD		ER	References
							Intra-day	Inter-day		
1	SPE GC-NPD	Diazinon	-	0.6	-	-	-	-	96	
		Chlorpyrifos	-	0.7	-	-	-	-	97	
	LLME GC-NPD	Diazinon	-	3.0	-	-	-	-	69	
		Chlorpyrifos	-	-	-	-	-	-	57	
2	CPE–back extraction/GC-FPD	Diazinon	0.1–20	0.04	0.12	0.9999	4.855	4.325	105.2	
3	SA-DSPE	Diazinon	2–1000	0.3	2	0.99	-	-	-	
4	SPE/HPLC	Diazinon	200–2000	200: 100	200: 150	0.999	-	-	81.76	
		Chlorpyrifos	200–2000	50–150	50–150	0.997	-	-	81.82	
5	SPE GC-IT-MS/S UHPLC-QqQ-MS/MS	Diazinon	0.1–100	0.022	0.073	0.996	6.3	11	94.6	
		Chlorpyrifos	0.1–100	0.010	0.034	0.995	8.6	19	90.6	
6	SFODME GC–MS	C	0.05–5.0	0.0048	0.0156	0.9968	-	-	0.1:100.3	
7	HS-SPME GC-NPD	Diazinon	10–120	2	-	-	-	4.9	100:9.2	
		Chlorpyrifos	10–120	10	-	0.9882	-	4.55	100:6.0	
8	DPX GC–MS	Chlorpyrifos	5.0–70	1.52	5.0	0.9979	6.5	10.2	91.6	
9	PP HPLC MS/MS	Chlorpyrifos	2–500	0.41	1.37	0.9976	3.8	6.05	85.5	
10	DLLME HPLC-DAD	Diazinon	500–4000	150	450	0.993	4.9	4.3	75.0–95.6	17
11	di-SPME GC-FPD	Diazinon	0.1 - 10	0.1	-	0.99	5.5	-	94.75	
		Chlorpyrifos	0.1 - 10	0.3	-	0.99	12.25	-	82	
12	GDME/GC–M–	Diazinon	0.01–100	0.0058	0.019	0.9988	1.46	1.83	99.02	This work
		Chlorpyrifos	0.01–100	0.016	0.054	0.9985	1.31	1.55	98.86	

For example, while the SPE method is generally less sensitive than GDME and has a more limited linear range, solvent-based methods such as LLME may also encounter environmental contamination and chemical waste disposal issues. On the other hand, GDME, with its lower solvent usage and smaller sample size, is recognized as a greener and more environmentally sustainable technique. Table 5 provides a detailed comparison of the performance of GDME/GC–MS with other extraction methods, demonstrating its significant superiority, particularly in terms of precision and reliability. For instance, while SPE and LLME methods may not offer reliable results due to their lower sensitivity and limited linear range, the GDME/GC–MS method, with a detection limit of 0.0058 µg/l for diazinon and 0.016 µg/l for chlorpyrifos, highlights its high efficiency in identifying and quantifying these compounds.

Moreover, the GDME/GC–MS method shows a high coefficient of determination (R²), indicating a strong correlation between analyte concentrations and chromatographic responses. This feature enhances the accuracy and validity of the results, allowing researchers to confidently use the obtained data. Ultimately, the extraction efficiency (ER) achieved with the GDME/GC–MS method was 99.0 % for diazinon and 98.9 % for chlorpyrifos, which is significantly higher than the extraction efficiencies of other methods listed in the table. For example, in SPE GC-NPD and LLME GC-NPD methods, the extraction efficiencies were reported as 96 % and 69 %, respectively. This indicates that the GDME/GC–MS method is capable of extracting the maximum amount of target analytes from the urine matrix.

In comparison, other methods such as SA-DSPE and DLLME also exhibit lower extraction efficiencies, implying that some of the analytes may be lost during the extraction process, potentially leading to false or unreliable results. Additionally, the high extraction efficiency of GDME helps minimize matrix interferences, ensuring that the results obtained from the samples more accurately reflect the true concentrations of the analytes. Furthermore, in methods such as HS-SPME GC-NPD and DPX GC–MS, where extraction efficiencies of 4.9 % and 10.2 % were reported, the reduced extraction efficiency may lead to higher costs for sample analysis and processing. In contrast, the high efficiency of GDME enhances the flexibility of the method for various laboratory conditions and industrial applications.

Overall, the high extraction efficiency of GDME allows for more effective use of subsequent analyses such as GC–MS, making the obtained information readily applicable for the identification and quantification of analytes. In general, these characteristics make GDME one of the best options for the extraction and analysis of target analytes in urine samples.

Given the multiple advantages of the GDME/GC–MS method, it can be concluded that this technique not only excels in terms of accuracy and sensitivity but also in environmental sustainability, making it a suitable choice for the extraction and analysis of organophosphorus compounds in biological samples. Table 5 shows results of previous studies that had used various sample preparation methods for the extraction of organophosphorous compounds and method validation parameters in comparison with obtained results.

## Conclusion

In this study, an innovative and sensitive method for the determination of organophosphorus pesticides, specifically diazinon and chlorpyrifos, in human urine samples was developed using Gas-Diffusion Microextraction (GDME) and Gas Chromatography-Mass Spectrometry (GC–MS). The results demonstrated that this method is capable of effectively and accurately extracting diazinon and chlorpyrifos from urine samples, with optimized extraction conditions determined through Box-Behnken design. The optimal conditions included an extraction time of 34 minutes, a temperature of 60°C, and a volume of 300 µL for the receiving solvent. Additionally, high temperature had a positive and significant impact on the extraction of diazinon, and 34 minutes was identified as the optimal time for achieving high yield. The GDME method, due to its unique features, including operational simplicity, low cost, reduced need for organic solvents, and the use of a gas-permeable membrane that prevents the entry of interfering components into the receiving solvent, was recognized as an effective and efficient sample preparation technique. These features led to cleaner extracts with less interference, and its ease of setup and use makes GDME an attractive and economical option for analyzing volatile and semi-volatile compounds. Calibration curves for diazinon and chlorpyrifos clearly demonstrated the high sensitivity of the GDME-GC–MS combination in measuring these compounds. The low detection limit and high precision of this method make it a suitable tool for monitoring and tracking these pesticides in biological samples. Comparison with other available methods showed that GDME offers significant advantages over traditional methods, such as simplicity, speed, reduced solvent consumption, and higher sensitivity. The use of the GDME-GC–MS method for the determination of organophosphorus pesticides in urine samples has been validated due to its outstanding benefits, including high accuracy, adequate sensitivity, ease of use, and reduced need for organic solvents. This method holds high potential for applications in various fields, such as environmental pollution monitoring, food quality control, and public health assessment. Furthermore, it could be employed in future studies for more precise monitoring and assessment of pesticide residues in biological samples and for the development of sample preparation methods.

## Limitations

This method may not provide accurate results under extreme conditions such as very high or low temperatures, or in more complex samples such as liver tissue and stomach contents. Therefore, researchers should consider appropriate experimental conditions for more complex samples.

## Ethics statements

This study was conducted in accordance with ethical guidelines and approved by the Ethics Committee of Kashan University of Medical Sciences (IR.KAUMS.REC.1402.012). All human volunteers provided informed consent regarding the purpose of the study and any potential risks associated with participation.

## CRediT author statement

**Mohammadreza Jafari:** Research conduct, data analysis, draft writing.

**Ali Gholami:** Research supervision, results review, draft revision.

**Maryam Akhgari:** Scientific advising, manuscript writing assistance.

## Declaration of competing interest

The authors declare that they have no known competing financial interests or personal relationships that could have appeared to influence the work reported in this paper.

## Data Availability

Data will be made available on request.
